# The Effect of Bimaxillary Orthognathic Surgery on Nasalance, Articulation Errors, and Speech Intelligibility in Skeletal Class III Deformity Patients

**DOI:** 10.29252/wjps.10.1.8

**Published:** 2021-01

**Authors:** Hamide Ghaemi, Erfan Emrani, Ali Labafchi, Khashyar Famili, Haleh Hashemzadeh, Sahand Samieirad

**Affiliations:** 1 Department of Speech Therapy, School of Paramedical Sciences, Mashhad University of Medical Sciences, Mashhad, Iran.; 2 Student Research Committee, Faculty of Dentistry, Mashhad University of Medical Sciences, Mashhad, Iran; 3 Department of Oral and Maxillofacial Surgery, Mashhad Dental School, Mashhad University of Medical Science, Mashhad, Iran.; 4 Department of Orthodontics, Tehran Dental School, Tehran University of Medical Science, Tehran, Iran.; 5 Oral and maxillofacial diseases research center, Mashhad University of Medical Sciences, Mashhad, Iran

**Keywords:** Bimaxillary, Orthognathic surgery, Nasalance, Articulation

## Abstract

**BACKGROUND:**

We aimed to detect the changes in nasalance, articulation errors, and speech intelligibility after bimaxillary orthognathic surgery in skeletal class III patients.

**METHODS:**

This double-blinded before and after quasi-experimental study was conducted in the Department of Maxillofacial Surgery, Qaem Hospital, Mashhad, Iran from Mar 2019 to Apr 2020. The main intervention was maxillary advancement with LeFort I osteotomy and mandibular setback surgery with bilateral sagittal split osteotomy (BSSO). The nasalance score, speech intelligibility, and articulation errors were evaluated one week preoperatively (T_0_), 1 and 6 months (T_1_, T_2_) postoperatively by a speech therapist. The significance level was set at 0.05 using SPSS 21.

**RESULTS:**

Eleven women (55%) and 9 men (45%) with a mean age of 31.95 ± 4.72 yr were enrolled. The mean maxillomandibular discrepancy was 6.15 ± 1.53 mm. The mean scores of nasalance for the oral, nasal, and oral-nasal sentences were significantly improved postoperatively (*P*<0.001). Pre-operative articulation errors of consonants /r/, /z/, /s/ and /sh/ were corrected following the surgery. The percentage of speech intelligibility was significantly increased over time (*P*<0.001).

**CONCLUSION:**

The patients might show a normal articulation pattern and a modified nasalance feature, following maxillary advancement plus mandibular setback surgery.

## INTRODUCTION

The lips, tongue, jaws, and velum work together to shape sounds into phonemes used in one’s language. The shaping of the vocal tract using the lips, tongue, jaw, and velum for speech output is called articulation; the lips, tongue, jaws, and velum are the articulators. ^1^^-^^4^ Assuming normal orofacial structures, individuals typically acquire the appropriate shaping of the vocal tract for the sounds of their language according to common developmental patterns entitled phonological development. ^1^^, ^^4^^, ^^5^

The consonants produced in any language can be described by their distinctive features as follows: 1) the place of production; 2) the degree of constriction between the two articulators; 3) the path of emission or energy enhancement, via the mouth versus the nose; and 4) the voicing, whether or not the vocal folds are in vibration. ^1^^, ^^5^^-^^7^

Individuals who have Class III malocclusion often present with difficulty producing labiodental and lingua-alveolar consonants. Furthermore, they tend to have difficulty with the lingua-alveolar consonants /t/, /d/, /l/, /n/, /s/, and /z/. These sounds should be produced with tongue-tip approximating the alveolar ridge. ^1^^, ^^6^^, ^^8^^-^^10^

Velopharyngeal dysfunction or insufficiency often co-occurs with malocclusion in individuals who have Class III malocclusion. ^1^^, ^^4^^, ^^5^^, ^^11^ Velopharyngeal dysfunction (VPD) is a generic term that describes a set of disorders resulting in the leakage of air into the nasal passages during speech production and articulation. Consequently, speech samples can demonstrate hypernasality, nasal emissions, and poor intelligibility. ^6^^, ^^12^^-^^18^ Therefore, it is important to differentiate these speech characteristics distinctly. 

One of the main surgical interventions for managing class III dentofacial deformities is mandibular setback surgery with or without maxillary advancement. ^2^^, ^^15^^, ^^19^^, ^^20^ Although orthognathic surgery enhances the masticatory function and facial esthetic, it has many impacts on speech characteristics. ^3^^, ^^10^^, ^^21^^, ^^22^

Bilateral sagittal split osteotomy (BSSO) and maxillary LeFort I osteotomy surgeries not only alters the relationship between the jaws and teeth but also affects the oral and pre-oral soft tissues, both of which can affect speech characteristics such as voice resonance and articulation quality. ^12^^-^^14^^, ^^16^^, ^^22^^-^^24^ Consequently, evaluating the voice changes after bimaxillary orthognathic surgery in non-syndromic class III patients seems obligatory.

Several studies have analyzed the multidimensional aspect of speech such as acoustic characteristics, voice resonance, and articulation pattern, as well as velopharyngeal dysfunction in patients with maxillofacial deformities and syndromes, especially cleft lip and palate disorders. ^3^^, ^^13^^-^^17^^, ^^21^ Mandibular setback surgery leads to remarkable changes in the upper airway dimensions. ^12^^, ^^18^^, ^^23^^, ^^25^ However, few studies investigated the impact of mandibular setback surgery on voice characteristics. ^2^^, ^^3^^, ^^19^^, ^^26^ Moreover, there is no universal agreement on the impact of bimaxillary orthognathic surgery on voice resonance and articulation in non-syndromic patients. ^2^^, ^^3^^, ^^19^^, ^^22^^, ^^23^^, ^^26^^, ^^27^

We aimed to detect the changes in voice nasalance, articulation errors, and speech intelligibility following bimaxillary orthognathic surgery (pure mandibular setback plus maxillary advancement) in skeletal class III deformity Persian speaker’s patients.

## MATERIALS AND METHODS

**Study design and patient selection**

This quasi-experimental study was approved by the Ethics Committee of Mashhad University of Medical Sciences (IR.MUMS.DENTISTRY.REC.1398.010). We followed the guidelines of CONSORT and the Helsinki Declaration. All patients signed an informed consent agreement before the study. 

This before-after experiment was carried out in the Department of Maxillofacial Surgery Qaem Hospital, Mashhad, Iran from Mar 2019 to Apr 2020. Inclusion criteria were all healthy (ASA I & II) non-syndromic Persian speaker patients aged 18 to 40 yr planned for bimaxillary orthogenetic surgery (Bimax) consist of pure mandibular setback surgery using bilateral sagittal split osteotomy (BSSO) plus pure maxillary advancement with LeFort I osteotomy. The subjects with a history cleft palate, velopharyngeal disorders, and history of dysphonia as well as hearing or perception problems and craniofacial syndromes were excluded.

The participants planned for genioplasty or maxillary inferior repositioning and impaction surgery impaction surgery were omitted from this analysis to remove potential confounding factors. Moreover, the patients with skeletal open bite, as well as who needed clock-wise or counter-clockwise occlusal plane rotation were excluded as well.

The type of deformities and surgical treatment plans and the age of the patients, sex, and the amounts of intraoperative mandibular and maxillary displacement (in millimeters) documented by the student under the supervision of the surgical staff in the checklist. Remarkably, the amount of pre-operative maxillomandibular discrepancy was measured clinically from the incisor tips. Besides, the clinical and cephalometric examinations determined the need for the mandibular setback plus maxillary advancement (Bimax) in our cases.

One speech therapist examined all patients’ voices before and after operations. Neither the examiner, who was a speech therapist nor the statistical analyst was aware of the surgical procedure. Nonetheless, the surgeon, the patients, and the student who completed the checklist were aware of the operation type. Therefore, it was a double-blinded study.

All of the patients underwent preoperative orthodontic decompensation. All orthognathic surgeries were conducted by the same surgeon and the same surgical team and hospital.

**Variables and Data recording**

The patients' age and gender were recorded. The authors observed and recorded the amount of skeletal movements of jaws during maxillary advancement and mandibular setback, intraoperatively. The amounts of mandibular setback displacement and maxillary advancement (in millimeter) were the study predictor variables. Besides, the nasalance score as well as speech intelligibility and articulation errors were the study outcome variables.

The evaluation and analysis of nasalance value, articulation errors and speech intelligibility were performed one week preoperatively (T0) and one month (T1) and six months (T2) postoperatively. All patients had orthodontic bracket appliances before and after surgery.

**Articulation**

The speech samples for the assessment of articulation were elicited using the phonetic picture naming test. This test requires subjects to name black and white drawings of common objects and actions. It elicits a speech sample containing instances of all Persian vowels and consonants in first, middle, and last positions of words. ^1^^, ^^4^^, ^^5^ For articulation analysis, consonant and vowel productions were compared with target productions and analyzed for error types at the segmental level. All analyses were based on a consensus narrow phonetic transcription made by the speech-language pathologist using the symbols and diacritics of the International Phonetic Alphabet. Only productions that were spontaneous naming of the stimulus pictures and of which the target was unmistakably clear were retained in the analysis. 

The speech sample to detect speech intelligibility contained 30 sec of spontaneous speech in response to open questions about the participants' hobbies and daily pastimes. The following formula was used to calculate the percentage of speech intelligibility: 

The number of correct words produced/the total number of words produced ×100. The speech intelligibility of 100% is ideal.

All voice recordings and analyses were made in an acoustic room at Qaem Hospital using a headset microphone (Shure SM 58, USA) and recorder (Sony ICD-PX240, Japan). A distance of 10 cm between the mouth and the microphone was set for each patient.

**Nasalance**

For the objective assessment of nasalance, the Nasometer was used. The Nasometer (model 6450, Kaypentax, USA), a microcomputer-based system manufactured by Kay Elemetrics13, was used for measurements of the nasalance values. Before initiating data collection, the Nasometer was calibrated in an acoustic room following the procedures outlined in the manual. Each subject was then asked to read three passages. The first passage contained ‘oral-nasal sentences’ (rainbow passage), covers 11.6% of nasal consonants as found in standard Persian speech. The second passage (Bahareh's Bag passage), as ‘oral sentences’, excludes nasal consonants and is normally used to detect hypernasality in a subject’s speech. The third passage was the ‘‘nasal sentences’’ (the text full of nasal consonants), loaded with nasal consonants and is designed to detect hyponasality in a subject’s speech. The validity and reliability of each of these three passages had been determined in Ghaemi and Memarian studies. ^11^^, ^^28^ If the subjects made a reading error, they were asked to read the passage again. Before testing, the Nasometer was calibrated and the Headgear was adjusted according to instructions provided by the manufacturer (Nasometer II model 6450, Kaypentax, New Jersey, USA).

Based on the evidence about the Nasalance analysis in Iranian populations, the normal range of Nasalance score for oral sentences was 19.43±7.45 and for nasal sentences was 67.15±5.67. ^7^^, ^^11^

**Data analysis**

The statistical analysis was carried out using SPSS version 21 (SPSS Inc., Chicago, IL). Qualitative variables were expressed as percentages, while the mean ± SD (standard deviation) was indicated for quantitative variables. The Shapiro-Wilk test was used to check and validate the data's normality. T-test was employed to compare the changes in the studied variables between males and females. The Pearson correlation test was applied to evaluate the relations between the variables. Moreover, the repeated measurements ANOVA test was used to compare the nasalance score, articulation error, and speech intelligibility analysis during the T0, T1, and T2. *P*-value<0.05 was considered statistically significant.

## RESULTS

Twenty individuals, including 11 females (55%) and 9 males (45%) with a mean age of 31.95±4.72 yr, and an age range of 21 to 40 yr participated. The mean discrepancy of maxillomandibular was 6.15±1.53 mm with a range of 4 to 8 mm. Moreover, the mean amounts of mandibular setback displacement and maxillary advancement were 3.30±0.86 mm and 2.85±0.74 mm, respectively. 

Fortunately, neither bad split nor inferior alveolar nerve injury was observed in our cases.

All individuals were evaluated at three-time intervals for voice nasalance score as well as articulation errors and speech intelligibility at three-time intervals (T0: one week preoperatively, T1: one month after surgery, T2: Six months postoperatively).

After bimaxillary orthognathic surgery, the mean changes in nasalance scores and speech intelligibility values between females and males were not significant (*P*>0.05) (Table 1). The Pearson's correlation coefficients showed no correlation between outcome variables and age, amounts of a preoperative maxillomandibular discrepancy, as well as mandibular setback displacement or maxillary advancement (Table 2). Table 3 depicts the mean of nasalance scores and speech intelligibility values in three-time intervals. The ANOVA test revealed that the mean nasalance score of oral and oral-nasal sentences decreased significantly in the postoperative time intervals (*P*<0.001). However, the mean nasalance score of nasal sentences increased significantly over time intervals (*P*<0.001). The percentage of speech intelligibility was increased significantly from 90.90% at T0 to 98.05% at T1 and increased to 100% at T2 (*P*<0.001) (Table 3). 

**Table 1:** Comparison of the mean changes in nasalance value and speech intelligibility between female and male patients after bimaxillary surgery

**Variables**	**gender**	**N**	**Mean ± SD**	***P*** value*
The mean nasalance value changes of oral sentence	Females	11	40.40 ±18.63	0.670
	Males	9	43.44 ±10.80	
The mean nasalance value changes of nasal sentence	Females	11	-14.96 ± 5.65	0.018
	Males	9	-21.31 ± 5.10	
The mean nasalance value changes of oral-nasal sentence	Females	11	25.09 ± 5.16	0.537
	Males	9	23.31 ± 7.45	
The mean change of Articulation intelligibility	Females	11	-10.70 ± 2.00	0.299
	Males	9	-9.33 ± 3.63	


*: T-test results

**Table 2:** The relations between changes in outcome variables and age, Maxillomandibular discrepancy, amount of mandibular setback and maxillary advancement

**Variables**		**Age**	**Maxillomandibular discrepancy**	**Amount of mandibular setback**	**Amount of maxillary advancement**
Nasalance value of oral sentences	Pearson's *r*	-0.324	-0.013	-0.044	-0.066
	*P*-value	0.163	0.957	0.854	0.782
Nasalance value of nasal sentences	Pearson's *r*	-0.153	0.147	0.187	0.108
	*P*-value	0.519	0.535	0.431	0.651
Nasalance value of oral-nasal sentences	Pearson's *r*	0.391	-0.182	-0.224	-0.138
	*P*-value	0.088	0.444	0.342	0.561
speech intelligibility	Pearson's *r*	0.139	-0.013	-0.015	-0.072
	*P-*value	0.559	0.957	0.949	0.763

**Table 3:** The mean values of nasalance value and speech intelligibility in pre- and postoperative time intervals

**Variables**	**Time intervals**	**N**	Mean ± SD **	**Range** **(Min-Max)**	***P*** -value*
Nasalance value of oral sentences (Hz)	T_0_	20	8.55^a^ ± 1.90	4 – 12	<0.001
	T_1_	20	7.30^b^ ± 1.49	4 – 9	
	T_2_	20	4.75^c^ ± 0.72	4 – 6	
Nasalance value of nasal sentences (Hz)	T_0_	20	69.10^a^ ± 3.61	64 – 78	<0.001
	T_1_	20	74.65^b^ ± 2.92	70 – 79	
	T_2_	20	81.25^c^ ± 2.97	75 – 86	
Nasalance value of oral-nasal sentences (Hz)	T_0_	20	38.80^a^ ± 2.63	33 – 44	<0.001
	T_1_	20	34.25^b^ ± 2.69	28 – 39	
	T_2_	20	29.30^c^ ± 2.15	25 - 33	
Speech intelligibility (%) ^$^	T_0_	20	90.90^a^ ± 2.38	87 – 96	<0.001
	T_1_	20	98.05^b^ ± 1.57	95 – 100	
	T_2_	20	100.00^b^ ± 0.00	100 – 100	


**Table 4:** Types and the number of articulation errors in pre- and postoperative time intervals.(n= 20 patients)

**Time intervals**	**Type of articulation error**	**Number (%) of patients with articulation error**
T_0_	distortion "z"	18 (90)
	distortion "r"	11 (55)
	distortion "s"	19 (95)
	distortion "sh"	19 (95)
T_1_	distortion "z"	2 (10)
	distortion "r"	6 (3)
	distortion "s"	1 (5)
	distortion "sh"	6 (3)
T_2_	articulation error	0 (0)


In this study articulations errors for all Persian consonant was evaluated in each of 20 patients. The various frequency of articulations errors, including /s/, /sh/, /z/ and /r/ were observed in patients. The highest number of articulation errors at T0 is related to /s/ and /sh/ letters, each with 19 cases. Followed by letter /z/ and /r/ with 18 and 11 cases, respectively. Moreover, the highest number of articulation errors at T1 is related to the letter /r/ and /sh/ each with 6 cases, followed by letter the letter "z" in 2 cases (Table 4). Interestingly, all the articulation errors corrected in T3 and its percentage was zero. 

## DISCUSSION

This before-after study aimed to evaluate the effect of bimaxillary orthognathic surgery, including mandibular setback with maxillary advancement on nasalance scores and articulation quality in patients with class III skeletal deformity. In this study bimaxillary orthogenetic surgery had a significant positive impact on the nasalance values probably due to modifying the structure and function of the velopharyngeal valve in the post-surgical condition. In other words, this surgery improved voice resonance features. Interestingly, all articulation errors were completely eliminated in postoperative tests. Subsequently, it led to complete and perfect speech intelligibility.

The effect of maxillary advancement with LeFort I osteotomy on articulation, resonance, velopharyngeal function, and voice was considered in many studies. ^12^^-^^14^^, ^^16^^, ^^22^^-^^24^ Nevertheless, studies examining the outcome of speech after BSSO with mandibular setback are limited, and contained contrary results. ^2^^, ^^3^^, ^^19^^, ^^26^ Moreover, the patient population and the surgical management in the above-mentioned studies were quite heterogeneous, without any attention to the type of surgery performed. ^2^^, ^^19^^, ^^22^^, ^^24^^, ^^26^ To the best of our knowledge, there was no agreement on the effects of orthognathic surgery on speech and voice. ^2^^, ^^3^^, ^^19^^, ^^22^^, ^^23^^, ^^26^^, ^^27^

Considering our research inclusion and exclusion criteria, the sample size was 20 patients, which was more than all similar studies, including Lee et al. ^9^ (nine class III patients), Sahoo et al. ^26^ (ten class III patients), Mishima et al. ^3^ (16 class III patients) and Ahn et al. ^2^ (eight class III patients). 

All assessments were performed before treatment (T0), one month after surgery (T1), six months after (T2), similar to Yavari et al. study. ^20^ It was not possible to evaluate one year follow-ups due to the COVID-19 pandemic. However, the results of this study were satisfactory and significant.

The patients who had a history of cleft palate, velopharyngeal and nasality disorders, and craniofacial syndromes, as well as who were candidates for genioplasty or maxillary impaction surgery or maxillary inferior repositioning were excluded from the present study to eliminate the additional confounding factor. Moreover, the patients who had skeletal open bite, or who needed clock-wise or counter-clockwise occlusal plane rotation were ecluded. The maxillary impaction and inferior repositioning may lead to compensatory clock-wise or counter-clockwise mandibular plan rotation, and the hyoid and tongue displacement consequently. ^2^^, ^^20^^, ^^24^^, ^^25^^, ^^29^ The above-mentioned factors might influence the voice production and articulation.

The Nasometer, as an instrumental assessment, is one of the most appropriate clinical tools for assessing and diagnosing nasalance problems. Nasometer was one of the most suitable instruments for evaluating resonance disorders in Persian populations. ^7^

Regarding the literature, surgical advancement of the maxilla, as was done in all of our patients, involves the anterior repositioning of the hard palate and the attached soft palate and velum. ^8^^, ^^21^ This forward movement of the hard palate may increase the anteroposterior dimensions of the nasopharynx and consequently alter the anatomical–topographical–functional relations of the velopharyngeal area. ^6^^, ^^9^^, ^^24^^, ^^30^ Velopharyngeal closure may, therefore, be compromised as a result of the increased distance the soft palate must move to close with the posterior pharyngeal wall completely during speech and subsequently may affect the resonance. ^2^^, ^^6^^, ^^24^^, ^^31^ Hyper-nasalization after maxillary advancement has always been a concern for maxillofacial surgeons. ^6^^, ^^13^^, ^^21^ On the other hand, the mandibular setback modulated the increase in the anteroposterior dimensions of the nasopharynx and caused the normal function of the velopharyngeal valves, and finally, we observed the normal nasalance score in patients. 

Based on our results, the mean nasalance score for nasal sentences was increased significantly over time, which in line with other studies. ^18^^,^^32^ The Nasalance score of oral sentences was changed significantly over time, decreased at each time interval compared to the previous time interval. This was in line with another study result. ^6^

The mandibular setback and maxillary advancement can improve the structure and function of the velopharyngeal valve. ^14^^, ^^23^^, ^^32^ Velopharyngeal valve closure was performed with a more natural quality to prevent airflow from entering the nose, and transferring to the mouth, during the articulation of oral consonants. ^5^^, ^^6^^, ^^13^^, ^^18^ On the other hand, during the articulation of nasal consonants, the velopharyngeal valve completely prevents airflow from entering the mouth and directs it into the nasal cavity with better quality than before. In other words, we observed more air emission from the nasal cavity for nasal consonants, and more air emission from the mouth for oral's consonants. ^6^^, ^^7^^, ^^11^^, ^^21^

Similar to oral sentences, the mean score of oral-nasal sentences also decreased significantly in postoperative intervals. Oral-nasal sentences include both groups of oral and nasal words. In oral-nasal sentences, the frequency of oral consonants was higher than in nasal consonants. ^4^^, ^^11^ Therefore, as mentioned earlier, the oral consonants became more oral after the surgical procedure, and a decrease in the nasalance score in the oral-nasal sentences has happened. 

In general, the facial skeleton (including the palate, maxilla, mandible, teeth, nasal cavity, etc.) directly affects the morphology of the resonant cavities of the vocal tract and the articulation of sounds. ^6^^, ^^18^ The forward movement of the maxilla leads to a change in airflow from a turbulent pattern to a suitable laminar and linear airflow pattern which causes the enhancement of voice resonance and nasalance. ^23^^, ^^25^

Mattos et al. documented the moderate reduction in oropharyngeal airway space following isolated mandibular setback surgery but a mild decrease in the oropharyngeal airway volume in the bimaxillary surgery. ^27^ Large amounts of mandibular setback may impede biological adaptation and significantly reduce the posterior airway space. The maxillary advancement concomitant with mandibular setback should be used in patients with class III malocclusion with severe anteroposterior discrepancy. ^33^

A rhinomanometry study revelead that maxillary advancement improved nasal airflow and voice resonance in bimaxillary surgery. ^21^ Similarly, a significant increase was found in nasopharyngeal airway space following bimaxillary surgery. ^32^

According to our study, phonetic test results at T0, all patients had similar type of articulation error which was distortion in sibilant sounds (/ s /, / sh /, / z /) and liquid sound / r /. At T1 and T2 the number and type of errors gradually decreased and so that at times T2, the errors were corrected entirely. This finding was consistent with other results. ^10^^,^^12^^,^^18^

The measurement of speech intelligibility was applied to show the process of improving articulation errors. The mean of speech intelligibility in our subjects was significantly increased over the postoperative intervals, which was consistent with another study's results. ^18^

Individuals who have Class III malocclusion often present with difficulty producing labiodental and lingua-alveolar consonants. These patients tend to have difficulty with the lingua-alveolar consonants /t/, /d/, /l/, /n/, /s/, and /z/. ^1^^, ^^6^^, ^^8^^-^^10^ They often produce this sound with the tip of the tongue contacting the maxillary incisors, called dentalization. This dentalization of the tongue tip alveolar fricatives /s/ and /z/ is usually described by listeners as a lisp or distortion. ^6^^, ^^8^^, ^^12^^, ^^18^^, ^^30^ The normal articulation of the /r/ consonant requires moving the tongue up and back toward the alveolar ridge and then beating it down and trilling. ^6^ In the present study, tongue was affected by the protruded position of mandible in the preoperative situation. Therefore, they had difficulty in trill movement of tongue and articulation. 

There are limited studies about the articulation errors analysis after orthognathic surgery. ^6^^, ^^9^^, ^^10^^, ^^18^ A distorted articulation of /r/ sound was improved following orthognathic surgery in class III patients. ^6^ It was in line with the present study findings. The speech changes of 20 patients was investigated who underwent surgery for the correction of various skeletal defects. The numbers of errors at the 3- and 6-month postoperative periods were lower than the preoperative numbers. ^10^ Vallino studied the articulation, voice, resonance, hearing sensitivity, and middle ear function of 34 patients who underwent orthognathic surgery. Thirty of the 34 patients showed speech errors before the surgery. The errors were mainly distortion of the sibilants /s/ and /z/. Articulation improved after surgery without speech therapy. Most of the preoperative articulation errors were eliminated 3 months after the surgery. ^18^ Nine Cantonese speakers undergoing osteotomy were studied for Class III skeletal deformity. The speech sample consisted of 6 words with the initial sibilant sound /s/. They reported a decrease in articulatory errors for the group after the surgery. ^9^

In preparation for orthognathic surgery, particularly when the patients, exhibit speech or resonance disorders, it is appropriate to have a speech-language pathologist evaluate the patient’s speech in order to identify patients who may be at risk for deterioration of speech articulation or resonance and airflow control following these procedures. ^6^^, ^^7^^, ^^18^^, ^^29^^, ^^31^

## CONCLUSION

The patients might show a modified articulation pattern (normal articulation) rather than the presurgical condition, following bimaxillary orthognathic surgery (maxillary advancement and mandibular setback with BSSO. The articulation errors were eliminated in patients at six months postoperatively. Moreover, bimaxillary orthognathic surgery might improve the nasalance scores in class III patients.

In preparation for orthognathic surgery, particularly when the patients’ exhibit speech or resonance disorders, it is appropriate to have a speech-language pathologist evaluate the patient’s speech to identify patients who may be at risk for deterioration of speech articulation or resonance and airflow control following these procedures. It is suggested to conduct more clinical trials with larger sample size, and long-term follow-ups in the future.

## Limitations and suggestions

Several important limitations need to be acknowledged in this research. First, the sample size was limited. Secon, the maxillomandibular skeletal discrepancies and jaw movements of our case were not large. Moreover, due to the COVID-19 pandemic in the world and Iran and quarantine protocols, it was not possible to follow up the patients for more than six months. Therefore, the authors suggest future researches with larger sample sizes, more skeletal discrepancies, and longer follow-ups.

The number of subjects was small, but our studied group was relatively homogenous, having the same deformity corrected by the same surgical procedure and operated by the same maxillofacial surgeon.

## References

[1] Dehqan A, Ansari H, Bakhtiar M (2010). Objective voice analysis of Iranian speakers with normal voices. J Voice.

[2] Ahn J, Kim G, Kim YH, Hong J (2015). Acoustic analysis of vowel sounds before and after orthognathic surgery. J Craniomaxillofac Surg.

[3] Mishima K, Moritani N, Nakano H, Matsushita A, Iida S, Ueyama Y (2013). Voice characteristics before versus after mandibular setback surgery in patients with mandibular prognathism using nonlinear dynamics and conventional acoustic analyses. J Craniomaxillofac Surg.

[4] Ghaemi H, Dehqan A, Mahmoodi-Bakhtiari B, Scherer RC (2020). Voice Changes During Pregnancy Trimesters in Iranian Pregnant Women. J Voice.

[5] Ghaemi H, Khoddami SM, Soleymani Z (2018). The Vocal Fold Dysfunction Questionnaire: Validity and Reliability of the Persian Version. J Voice.

[6] Van Lierde KM, Schepers S, Timmermans L, Verhoye I, Van Cauwenberge P (2006). The impact of mandibular advancement on articulation, resonance and voice characteristics in Flemish speaking adults: a pilot study. Int J Oral Maxillofac Surg.

[7] Morteza F, Akbar D, Mina F, Hashem S, Seyed Habibollah K (2016). Determining Normative Nasalization Scores Among Persian-speaking Adults. Journal of Modern Rehabilitation.

[8] Vallino LD, Tompson B (1993). Perceptual characteristics of consonant errors associated with malocclusion. J Oral Maxillofac Surg.

[9] Lee AS, Whitehill TL, Ciocca V, Samman N (2002). Acoustic and perceptual analysis of the sibilant sound/s/before and after orthognathic surgery. J Oral Maxillofac Surg.

[10] Ruscello DM, Tekieli ME, Jakomis T, Cook L, Van Sickels JE (1986). The effects of orthognathic surgery on speech production. Am J Orthod.

[11] Ghaemi H, Sobhani Rad D, Khodadoost M, Elyasi M, Mardani N (2015). Detecting Normal Values of Nasalance Scores in 7-11-Year-old Boys. Journal of Paramedical Sciences & Rehabilitation.

[12] Dalston RM, Vig PS (1984). Effects of orthognathic surgery on speech: a prospective study. Am J Orthod.

[13] Hagberg E, Flodin S, Granqvist S, Karsten A, Neovius E, Lohmander A (2019). The Impact of Maxillary Advancement on Consonant Proficiency in Patients With Cleft Lip and Palate, Lay Listeners' Opinion, and Patients' Satisfaction With Speech. Cleft Palate Craniofac J.

[14] Janulewicz J, Costello BJ, Buckley MJ, Ford MD, Close J, Gassner R (2004). The effects of Le Fort I osteotomies on velopharyngeal and speech functions in cleft patients. J Oral Maxillofac Surg.

[15] Kim SK, Kim JC, Moon JB, Lee KC (2012). Perceptual speech assessment after maxillary advancement osteotomy in patients with a repaired cleft lip and palate. Arch Plast Surg.

[16] McComb RW, Marrinan EM, Nuss RC, Labrie RA, Mulliken JB, Padwa BL (2011). Predictors of velopharyngeal insufficiency after Le Fort I maxillary advancement in patients with cleft palate. J Oral Maxillofac Surg.

[17] O'Gara M, Wilson K (2007). The effects of maxillofacial surgery on speech and velopharyngeal function. Clin Plast Surg.

[18] Vallino LD (1990). Speech, velopharyngeal function, and hearing before and after orthognathic surgery. J Oral Maxillofac Surg.

[19] Jorge TM, Brasolotto AG, Gonçales ES, Filho HN, Berretin-Felix G (2009). Influence of orthognathic surgery on voice fundamental frequency. J Craniofac Surg.

[20] Yavari N, Samieirad S, Labafchi A, Rezaeetalab F, Eshghpour M (2020). Is There an Increase in the Risk of Obstructive Sleep Apnea After Isolated Mandibular Setback Surgery? An Evaluation Using the STOP-BANG Questionnaire. J Oral Maxillofac Surg.

[21] Pourdanesh F, Sharifi R, Mohebbi A, Jamilian A (2012). Effects of maxillary advancement and impaction on nasal airway function. Int J Oral Maxillofac Surg.

[22] Tatli U, Surmelioglu O, Tukel HC, Kurkcu M, Benlidayi ME (2020). Effects of Orthognathic Surgery on Voice Characteristics. J Oral Maxillofac Surg.

[23] Chen F, Terada K, Hua Y, Saito I (2007). Effects of bimaxillary surgery and mandibular setback surgery on pharyngeal airway measurements in patients with Class III skeletal deformities. Am J Orthod Dentofacial Orthop.

[24] Hassan T, Naini FB, Gill DS (2007). The effects of orthognathic surgery on speech: a review. J Oral Maxillofac Surg.

[25] Demetriades N, Chang DJ, Laskarides C, Papageorge M (2010). Effects of mandibular retropositioning, with or without maxillary advancement, on the oro-naso-pharyngeal airway and development of sleep-related breathing disorders. J Oral Maxillofac Surg.

[26] Sahoo NK, Roy ID, Kulkarni V (2019). Mandibular setback and its effects on speech. Oral and Maxillofacial Surgery Cases.

[27] Mattos CT, Vilani GN, Sant'Anna EF, Ruellas AC, Maia LC (2011). Effects of orthognathic surgery on oropharyngeal airway: a meta-analysis. Int J Oral Maxillofac Surg.

[28] Memarian A, Ghorbani A, Torabi Nejad F, Keyhani MR (2010). Designing a Farsi text for the assessment of adult voice features and determining its validity and reliability in measuring the fundamental frequency and intensity of speech. Journal of Research in Rehabilitation Sciences.

[29] Niemi M, Laaksonen JP, Peltomäki T, Kurimo J, Aaltonen O, Happonen RP (2006). Acoustic comparison of vowel sounds produced before and after orthognathic surgery for mandibular advancement. J Oral Maxillofac Surg.

[30] Wakumoto M, Isaacson KG, Friel S (1996). Preliminary study of articulatory reorganisation of fricative consonants following osteotomy. Folia Phoniatr Logop.

[31] Ward EC, McAuliffe M, Holmes SK, Lynham A, Monsour F (2002). Impact of malocclusion and orthognathic reconstruction surgery on resonance and articulatory function: an examination of variability in five cases. Br J Oral Maxillofac Surg.

[32] Cakarne D, Urtane I, Skagers A (2003). Pharyngeal airway sagittal dimension in patients with Class III skeletal dentofacial deformity before and after bimaxillary surgery. Stomatologija.

[33] Hasebe D, Kobayashi T, Hasegawa M (2011). Changes in oropharyngeal airway and respiratory function during sleep after orthognathic surgery in patients with mandibular prognathism. Int J Oral Maxillofac Surg.

